# Sensitivity of *Anopheles gambiae *population dynamics to meteo-hydrological variability: a mechanistic approach

**DOI:** 10.1186/1475-2875-10-294

**Published:** 2011-10-10

**Authors:** Gianni Gilioli, Luigi Mariani

**Affiliations:** 1 University of Brescia, Medical School, Department of Biomedical Sciences and Biotechnologies, Viale Europa 11, I-25123 Brescia, Italy; 2 University of Milan, Department of Plant Production, Via Celoria 2, 20133 Milano, Italy; 3 CASAS (Center for the Analysis of Sustainable Agricultural Systems), 37 Arlington Ave., Kensington, CA 94707, USA

## Abstract

**Background:**

Mechanistic models play an important role in many biological disciplines, and they can effectively contribute to evaluate the spatial-temporal evolution of mosquito populations, in the light of the increasing knowledge of the crucial driving role on vector dynamics played by meteo-climatic features as well as other physical-biological characteristics of the landscape.

**Methods:**

In malaria eco-epidemiology landscape components (atmosphere, water bodies, land use) interact with the epidemiological system (interacting populations of vector, human, and parasite). In the background of the eco-epidemiological approach, a mosquito population model is here proposed to evaluate the sensitivity of *An. gambiae *s.s. population to some peculiar thermal-pluviometric scenarios. The scenarios are obtained perturbing meteorological time series data referred to four Kenyan sites (Nairobi, Nyabondo, Kibwesi, and Malindi) representing four different eco-epidemiological settings.

**Results:**

Simulations highlight a strong dependence of mosquito population abundance on temperature variation with well-defined site-specific patterns. The upper extreme of thermal perturbation interval (+ 3°C) gives rise to an increase in adult population abundance at Nairobi (+111%) and Nyabondo (+61%), and a decrease at Kibwezi (-2%) and Malindi (-36%). At the lower extreme perturbation (-3°C) is observed a reduction in both immature and adult mosquito population in three sites (Nairobi -74%, Nyabondo -66%, Kibwezi -39%), and an increase in Malindi (+11%). A coherent non-linear pattern of population variation emerges. The maximum rate of variation is +30% population abundance for +1°C of temperature change, but also almost null and negative values are obtained. Mosquitoes are less sensitive to rainfall and both adults and immature populations display a positive quasi-linear response pattern to rainfall variation.

**Conclusions:**

The non-linear temperature-dependent response is in agreement with the non-linear patterns of temperature-response of the basic bio-demographic processes. This non-linearity makes the hypothesized biological amplification of temperature effects valid only for a limited range of temperatures. As a consequence, no simple extrapolations can be done linking temperature rise with increase in mosquito distribution and abundance, and projections of *An. gambiae *s.s. populations should be produced only in the light of the local meteo-climatic features as well as other physical and biological characteristics of the landscape.

## Background

Space and time variability of climate and more generally of environmental variables are expected to affect the morbidity and mortality pattern of human and animal diseases [1-3] with particular emphasis on vector-borne infections [4,5]. Among the vector-borne human diseases, malaria has the potential to modify the area of distribution and the epidemic pattern in response to space-time variation of temperature and rainfall, due to the role of these meteorological variables on the ecology and the behaviour of the vectors as well as on their environment [6].

Temperature affects malaria transmission in various ways [7,8], influencing, for example, the sporogonic period of the *Plasmodium *parasite, the developmental period of the aquatic stages of the vector and the fecundity of the adults. Most of the studies on the effects of temperature on malaria has been carried out with methods that can be referred to statistical ecological models and to semi-quantitative methods or simplified dynamical models based on indexes like the basic reproductive rate. For example, attempts have been made to interpret recent and anomalous increases in malaria prevalence as the consequence of temperature trends in the tropics. Different quantitative approaches have demonstrated the role of temperature changes [9,10] or thermal-pluviometric variability associated to the El Niño-Southern Oscillation (ENSO) [11,12] in the malaria resurgence in East African highlands.

Rainfall affects malaria acting not only on persistence of water bodies but also on physical and bio-chemical characteristics of aquatic environments hosting the pre-imaginal stages of mosquito vectors. Heavy rains and related floods are reported to cause the major malaria outbreaks in semi-arid or arid lowlands [13], whilst spatial and temporal variations in rainfall have been proved to determine the nature and scale of malaria transmission in highland areas. Abnormal rainfall events have been shown to precipitate malaria epidemics even in wetter areas, as evidenced by epidemics in Uganda, Kenya and Ethiopia [14]. Global circulation patterns have been correlated to malaria prevalence, as for the influence of El Niño Southern Oscillation (ENSO) in Uganda [11,15] and for the effect of the Indian Ocean Dipole (IOD) on malaria risk in the East African Highlands [12].

Epidemiological research on relationship between climate variability and human health has been far long dominated by a risk factor analysis founded on an empirical approach. This latter obeys to the *ceteris paribus *logic [16] focusing on impacts of single (e.g., temperature) or several variables (e.g., rainfall and temperature). In such framework, climate variability is interpreted as a risk factor acting on some transmission parameters. This gives rise to persuasive because intuitive conclusions, but also prone to the risk to ignore several key factors for the transmission and epidemiology of the diseases [8]. Examples are the increased vectorial capacity (due to natural or man-made causes), the immigration of infected people to receptive areas, the immigration of non-immunes to endemic areas, and drug resistance which have been identified as the major epidemic precipitating factors [17]. Also economic and social factors combine to bring about the response of health systems to climate variability [17-19].

In eco-epidemiological studies [20] empirical or statistical oriented modelling approaches provide useful insight when the relationships within the systems are insufficiently known. However, mechanistic approaches can be more profitable when the underlying functional mechanisms of the processes of interest are known in sufficient detail. More specifically, mechanistic models are crucial to evaluate the role of biological, ecological and socio-economic processes in health systems dynamics [20], characterized by complexity and non-linearity acting on a wide range of space and time scales [21,22]. Hence, mechanistic models can be usefully applied to the analysis and integrated assessment of vector-borne diseases in order to simultaneously take into account the influence of physical and biotic ecosystem components on the disease dynamics [23,24]. This approach is also relevant to understand the climatic effects on waterborne and vector-borne diseases and to provide indications for a proper development of ecological and epidemiological models [8].

In order to adopt a mechanistic approach, an improved inferential reasoning in disease ecology is required. Following [16] this implies the use of complex causal diagrams and methods based on triangulation among field data, experiments, and modelling able to understand and anticipate complex and nonlinear dynamics of eco-epidemiological systems [20]. The causal inference in the eco-epidemiology of malaria necessitates the development of new methods that could overhaul limits in the traditional statistical methods based on hypothesis testing and correlation structure. Following Reiter's comments [8], approaches with simplified mathematical models to malaria epidemiology can lead to incorrect interpretation of the effects of environmental forcing variables on the epidemic process. Even considering the population dynamics of the vectorial component of the malaria system only, integrative processes that give rise to counter-intuitive system behaviour responses may occur. As a consequence, the emerging population dynamical patterns are not simply interpretable in terms of linear effect of environmental forcing variables. Also traditional methods, not referred to process-based models, have shown that environmental forcing variables (e.g., temperature) act on the mosquito dynamics giving rise to an unexpected complexity and no simple conclusions can be drawn [8,25-28].

On the basis of these assumptions, the impact of climate variability on one of the component of the malaria system, the vector mosquito, is here analysed. To this aim a physiologically based model [29] is used to investigate the impact of specific patterns of change in meteorological and hydrological variables on the *Anopheles gambiae *s.s. population dynamics at microscale [30]. Three process-based models have been developed and used to represent the interaction among meteorological, hydrological, and vector subsystems. In particular, a meteorological sub-model produces spatial fields of air temperature and rainfall, a hydrological sub-model describes water level and temperature in immature mosquito habitats, and a mosquito vector sub-model describes the structured population dynamics of *An. gambiae *s.s. considering variation in both immature and adult abundance. The model, hereafter denoting the ensemble of the three process based sub-models, is used to perform a sensitivity analysis over a set of four Kenyan sites, representing four different eco-epidemiological conditions. Results are then evaluated in order to derive general pattern of vector mosquito population responses to climate variability.

## Methods

### Model components

The analysis of the mosquito vector population system is based on an ecological framework that considers all the relevant landscape components and their influence on the mosquito population dynamics. In the model the landscape comprises a spatially defined portion of land characterized by physical, ecological and human elements, including land-use [31]. All these elements are viewed in their capacity to create suitable conditions for the establishment and persistence of a mosquito population. Essential parts of the landscape are represented by land cover (crops, natural vegetation, buildings, etc.), atmosphere (surface weather variables), soil (textural and hydrologic parameters) and hydrosphere (ephemeral or perennial water bodies that are potential breeding sites for mosquitoes). The model also considers a stable human host population as source of blood for the mosquito adults reproduction.

### Meteorological determinants influencing mosquito population dynamics

As discussed by [32], mid-latitude areas that in recent centuries were widely affected by malaria, benefited in the last decades from public health policies that have limited the problem to tropical rainy areas [8]. By consequence this paper focuses on tropical climates (Koeppen's A - [33]) with particular reference to Kenya, a country which sits astride the Equator and where performance of the model has been evaluated for four representative sites. Additional file 1 allows to interpret factors influencing the meteorological variables of major importance for mosquito population like air and water temperature and rainfall. Temperature depends on processes occurring at microscale (e.g. surface energy balance, breeze circulations), mesoscale (e.g. tropical disturbances) and macroscale (e.g. Hadley cell, Enso, Madden-Julian Oscillation, monsoons). Macro and mesoscale precipitation systems are mainly fed by humidity produced by microscale phenomena acting into the boundary layer. This link among scales is at the root of a strong space and time variability detectable in the whole set of surface variables (first of all cloud coverage and precipitation, radiation fluxes, air temperature and humidity) that characterizes the tropical environments and affects the dynamics of mosquito population. The adoption of conceptual schemes and monitoring tools (real-time meteorological networks, remote sensing) able to account for this variability is of paramount importance to improve the risk mapping methods and the early warning systems [34].

### Hydrological determinants

Ephemeral and perennial pools occur commonly throughout the tropical areas where represent unique or preferential habitats for a lot of species that support a rich invertebrates community. The physical-chemical characteristics and the temporal persistence of water bodies surface (hydro-period) affect the composition and reproduction of vegetation and fauna and drive the establishment and the maintenance of the *Anopheles *spp. aquatic phases [35,36]. Additional file 2 shows the main hydrological processes influencing larval habitats.

### Scale aspects and landscape characteristics

Meteorological variables are fundamental driving variables for the malaria vectors population dynamics acting not only directly on vectors but also indirectly as determinants of the environmental heterogeneity in general and hydrological features in particular (e.g., presence and characteristics of water bodies at macro, meso and microscale). Heterogeneity and scale aspects related to landscape and environmental forcing variables have to be adequately addressed to develop appropriate models of mosquito population dynamics. Meteorological and hydrological variables are respectively described in a scale perspective by [37] and [34]. As far as meteorological variables are concerned, a monitoring approach based on a network of stations at inadequate scale could give rise to an inaccurate analysis and simulation of mosquito population dynamics. The existing mean density of one weather station per 26,000 km^2 ^for the African Continent, as reported in [38], gives a quite inaccurate description of the spatial distribution of precipitation, with strong consequences on the accuracy of hydrological analysis. An integrated monitoring approach exploiting both surface stations and remote sensed data (e.g., satellite operating in the regions of thermal infrared, visible and microwaves) could at least partially overcome the above-mentioned limitation.

Scale considerations are also important for the mosquito populations. The pre-imaginal phase of malaria vectors presents a very reduced mobility of the order of 10^-1 ^m day^-1^. Vice-versa mosquito adults are often characterized by a high mobility, of the order of 10^2 ^m day^-1^. The same or higher mobility applies to human hosts. These aspects impose the selection of an adequate resolution in order to model the interactions between environment, mosquito and human host.

The above-mentioned multi-scale variability justifies a multi-scale approach [39] based on a lattice model with a grid defining square spatial units or cells of different dimension according to the level of resolution. A proposal of scale classification of phenomena involved in the malaria vector population dynamics system at different spatial and temporal resolution is summarized in Additional file 3. Three levels of resolution are considered, respectively for spatial units of 1 × 1 km (micro-scale), 10 × 10 km (meso-scale) and 100 × 100 km (macro-scale). These three levels can be also associated to different levels of management, where policies are mostly directed by macro-scale analysis, strategies by meso-scale, and tactics by micro-scale.

### Model structure

#### General architecture

**Figure 1:**
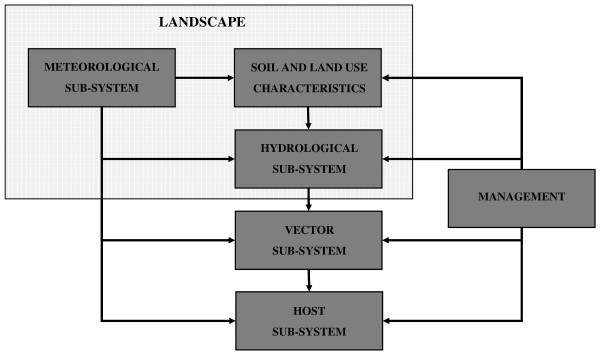
**Mosquito vector population system**. Conceptual scheme reporting the components (sub-systems) of the mosquito vector population system and their interactions.

**Figure 2:**
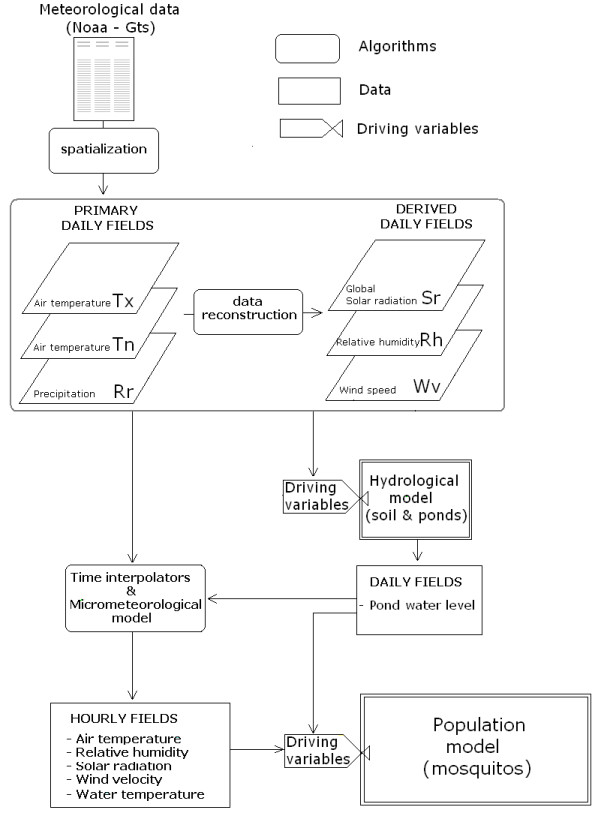
**General scheme of the model**. The scheme focuses on meteorological sub-model which fed hydrological and mosquito vector sub-models. Both hourly and daily loop are shown. Rectangular boxes are for state variables, ellipses for driving variables and faucets for rate ones.

For the objective of the analysis here performed a population dynamics model has been developed in the light of the flux diagram in Figure 1 considering the interaction of the landscape and the vector components at a micro-level spatial resolution (spatial unit of 1 × 1 km). For human host a constant population density is adopted and no management actions are assumed to be undertaken. The general scheme of the model, represented in Figure 2, follows the basic framework of dynamical modelling [40], and specific sub-models for different sub-systems are developed and used. Two loops with different time steps coexist in the simulation model. The slow loop is used by models with a daily time step (meteorological and hydrological models), the fast loop by models with an hourly time step (vector model and water temperature models). The model is driven by gridded daily fields of meteorological data (global solar radiation, maximum and minimum relative humidity, average wind speed) derived from meteorological stations data. Daily weather variables are used to drive hydrological models (daily water balance of soils and larval habitats) and to produce hourly values of meteorological data, which in turn drive mosquito population model.

#### Meteorological data and model

A daily 1983-2009 meteorological dataset collected by NOAA-Gsod [41] and referred to synoptic stations of Kenya (Additional file 4), has been adopted as meteorological input for the model. A preliminary check of the entire dataset has shown the lack of 40.6% of maximum temperature data (*Tx*), 39.4% of minimum temperature (*Tn*), and 40.4% of total precipitation (*Rr*). The quality of the dataset for the four stations adopted for the sensitivity analysis has been evaluated matching yearly means of the available values with yearly mean values for the same stations reported by the FAO Climwat database [42] (see Table 1). The strict agreement between the two sources testifies the good quality of NOAA-Gsod data.

**Table 1:** Yearly mean climatic features for the four selected locations.

Feature	Unit	Reference sites			
		**Nairobi**	**Nyabondo**	**Kibwezi**	**Malindi**

**mean altitude**	m a.s.l.	1778	1532	904	5

**location**		Highlands east of the Rift Valley	Area around the Lake Victoria	Arid high ground	Southern Coastal Area

**Reference station**		Nairobi Airport	Kisumu	Marsabit	Malindi airport

**Reference station height**	m a.s.l.	1624	1146	1345	20

***Tx***	°C	24.0	26.8	27.9	30.6

***Tn***	°C	13.6	14.5	18.1	23.3

***Td***	°C	18.8	20.7	23	26.9

**Stdev_*Td***	°C	2.0	1.5	1.5	1.7

***Dtr***	°C	10.5	12.3	9.8	7.2

***Rr***	mm	796.4	1285.5	712.2	889.3

***Ev***	mm	1882	2156	2033	1900

Rh*	%	77.7	66.6	84.6	84.3

Sr*	MJ m^-2^	6942	7422	6828	7866

Sh*	h	2457	2787	2394	3069

Wt*	km year^-1^	55500	38880	74430	73410

**Climate**		Tropical climate modified by highlands	Equatorial climate modified by lake Victoria	Semiarid to arid climate	Modified equatorial climate of the Coast

Data referred to the reported reference meteorological station and climatic classification from Ininda et al. (2007). Tx, Tn, Td = maximum, minimum and mean temperature; Dtr = daily thermal range; Ev = Evaporation; Rh = Relative humidity; Sr = Solar radiation; Sh = sunshine duration; Wt = wind totalized. Tx, Tn, Td, Dtr and Rr are from 1983-2009 data (NOAA, 2010). Data from FAO Climwat database[54] are highlighted with the asterisks.

Unknown Tx, Tn and Rr data for reference grid points are obtained applying to stations data a weighted mean with weight inversely proportional to the square of the distance (Inverse Distance Weighting Mean - IDWM) [43]. In order to apply IDWM method, temperature data have been previously homogenized to the same height and aspect of the unknown grid point adopting specific altitude and aspect gradients [44]. The same IDWM method has been adopted to rebuild unavailable weather stations data.

For the purpose of this paper the model has been applied to two different digital terrain models (DTM): the USGS Gtopo30, with pixel of about 1,000 × 1,000 m [45], and the NASA SRTM mission, with a resolution of about 100 × 100 m [46]. Hourly air temperature data (*T_a_*), obtained applying the Parton and Logan model [47] to daily data (*Tx *and *Tn*), feed an energy balance based model [48] producing hourly water temperature of larval habitats (*T_w_*)

$$T_{w}=T_{a}+\left(R_{n}+G+L\times E\right)∕h_{u}$$

where *R_n _*is the net radiation resulting from the surface radiation balance, *G *is the heat stored into the water, *L *× *E *is the latent heat flux and *h_u _*is the sensible heat transfer coefficient. The water temperature model is described in detail in the Additional file 2.

#### Hydrological model

**Figure 3:**
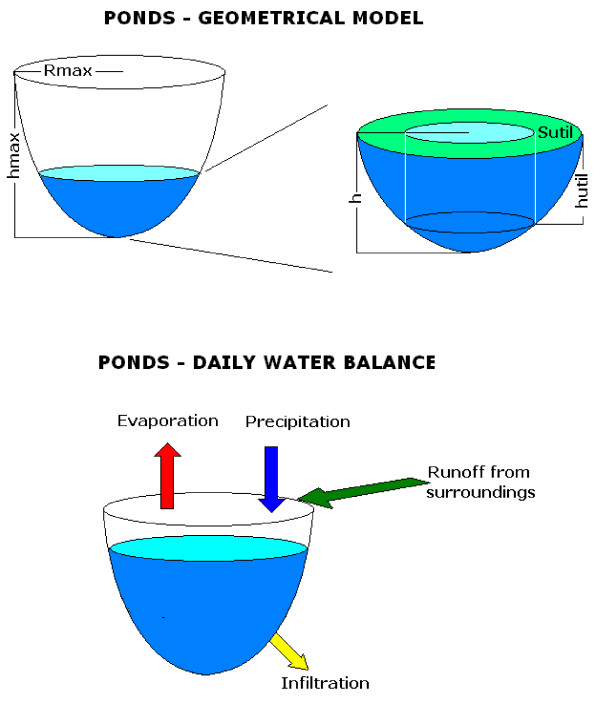
**Geometrical model of the larval habitats**. Larval habitats are represented as paraboloids of revolution inscribed in a parallelepiped with a squared base.

The hydrological model that describes the state of water bodies follows the classical approach based on the continuity equation (conservation of water) applied to a suitable reservoir as described for example by Hood et al. [49]. The water inflow is due to rainfall and runoff from the surroundings while the outflow is due to evaporation from water surface and seepage from the bottom of the water body. The model works with a daily time step and simulates the water content of five classes of water bodies generated by a suitable paraboloid of rotation (Figure 3). The final result is a quantitative estimate of the length of the hydro-period under different weather and hydrological conditions. The adoption of a single geometric solid to approximate different larval habitats is useful in order to create a general framework where the physical processes that affect the behaviour of water bodies can act. In the model, the runoff is simulated with a curve number model, evaporation is simulated with the Hargreaves and Samani evapotranspiration equation applying a suitable correction factor and seepage is simulated with the Morel - Seidoux approach. The hydrological algorithm, the calibration and validation processes and the final model parameterization and the references are reported in the Additional file 2.

#### Mosquito vector model

**Figure 4:**
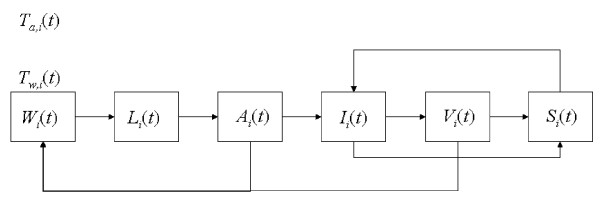
**Flux diagram representing the mosquito vector population dynamics**. The population stages and their interaction with water bodies, atmosphere and human hosts are indicated.

The vector model here used has been developed by [30] for the simulation of the stage-structured mosquito population dynamics. The model adopts a physiologically-based approach where bio-demographic rate functions depends on environmental forcing variables (e.g., temperature), and on the ratio between resources availability and per-capita demand for resources [29]. For a given spatial unit *i*, the model simulates the variation in the abundance of mosquito immature stages *L_i _*(*t*), non-infected *A_i _*(*t*) and infected *V_i _*(*t*) adult stage (Figure 4). The abundance in immature and adult mosquito stages varies according to stage-specific bio-demographic and behavioural (i.e., search for resources, both larval habitats and human hosts) processes. More in detail (*i*) immature developmental rate depends on local water temperature (*T_w, i _*(*t*)), (*ii*) survival rate of immature depends on local water temperature *T_w, i _*(*t*), the availability of water bodies surface *W_i _*(*t*), structured in many different dimensional classes, and age, (*iii*) survival rate of the adults depends on air temperature *T_a, i _*(*t*) and age, (*iv*) fecundity rate of adults depends on air temperature *T_a, i _*(*t*), age, availability of blood and water resources (*W_i _*(*t*)), as well as on behavioural process of search for those resources (functional responses). The population of human hosts in the cell *i *is structured in susceptible *S_i _*(*t*), infected *I_i _*(*t*) and recovered *R_i _*(*t*). For the purpose of this work the vector model is simplified, and all processes related to the transmission and development of infection in the vector as well as in the host are neglected. As a consequence, mosquito adults are grouped into a single category *M_i _*(*t*) = *A_i _*(*t*) + *V_i _*(*t*), and the same has been done for the human population, setting a constant number of individuals per cell.

#### Model parameterization and sensitivity analysis

Calibration, validation and sensitivity analysis are crucial to obtain a model useful for operational purposes [50]. Sensitivity analysis has been applied to the calibrated hydrological, meteorological and vector models to evaluate the forcing role of climate variability on the dynamic of *An. gambiae *s.s. population in four different Kenyan sites. Sensitivity analysis for a system driven by meteorological variables can be carried out imposing a perturbation to the time series of weather data by means of stochastic methods or adopting deterministic methods modifying the dataset with predefined changing factors [51]. The latter method is here adopted, imposing progressive changes to the daily time series of temperature and precipitation.

The observational time series of temperature *T *(*S*) and rainfall *R *(*S*) are considered the reference standard. For the sensitivity analysis an additive coefficient *h *(with *h *= -3, -2, -1, 0, +1,+2, +3) has been applied to daily temperature *T *(*S*) obtaining the series [*T *(*S*) - 3 = *T*(-3), *T *(*S*) -2 = *T*(-2), *T *(*S*) -1 = *T*(-1), *T *(*S*) + 0 = *T*(0), *T *(*S*) + 1 = *T *(+1), *T *(*S*) + 2 = *T*(+2), *T *(*S*) + 3 = *T*(+3)]. A multiplicative coefficient *k *(with *k *= 0.8, 0.9, 1, 1.1, 1.2) has been applied to daily rainfall obtaining the series [0.8*R*(*S*) = *R*(0.8), 0.9 *R*(*S*) = *R*(0.9), 1*R*(*S*) = *R*(1),1.1 *R*(*S*) = *R*(1.1),1.2 *R*(*S*) = *R*(1.2)].

The seven series of temperature and the five series of precipitation were associated in a factorial design in order to obtain a total of 35 combinations or meteorological scenarios. These scenarios have been used to evaluate the impact of climatic variability on the mean abundance of mosquitoes expressed as the total number of immature and adults per spatial unit. Simulation have been performed at micro-scale, considering a single 1 × 1 km cell, and for the period 1983-2009. Values reported in the Additional file 2 are used as initial conditions for the number of larval habitats per cell referred to the five dimensional classes, for the number of mosquito immature/larval habitat in each class of larval habitat, and for the total constant human population per cell.

The mean value of the simulated abundance of immature $\bar{L}\left(T\left(S\right),R\left(S\right)\right)$ and adult $\bar{M}\left(T\left(S\right),R\left(S\right)\right)$ stages are obtained using the reference time series for the period 1983-2009.

Let be $\bar{M}\left(T\left(S\right)+h,kR\left(S\right)\right)$ and $\bar{L}\left(T\left(S\right)+h,kR\left(S\right)\right)$ the mean values of adults and immature abundance for a modified temperature and precipitation regimes, then the indexes of variation of adults $P_{M}=\bar{M}\left(T\left(S\right)+h,kR\left(S\right)\right)∕\bar{M}\left(T\left(S\right),R\left(S\right)\right)$ and immature $P_{L}=\bar{L}\left(T\left(S\right)+h,kR\left(S\right)\right)∕\bar{L}\left(T\left(S\right),R\left(S\right)\right)$ population abundance have been calculated for each weather regime.

## Results

### Site-specific sensitivity analysis

The obtained results for four different sites are hereafter presented. All changes in vector population abundance are compared to the simulated abundance obtained for the observational time series 1983-2009 (reference condition *T*(0) and *R*(1)).

**Figure 5:**
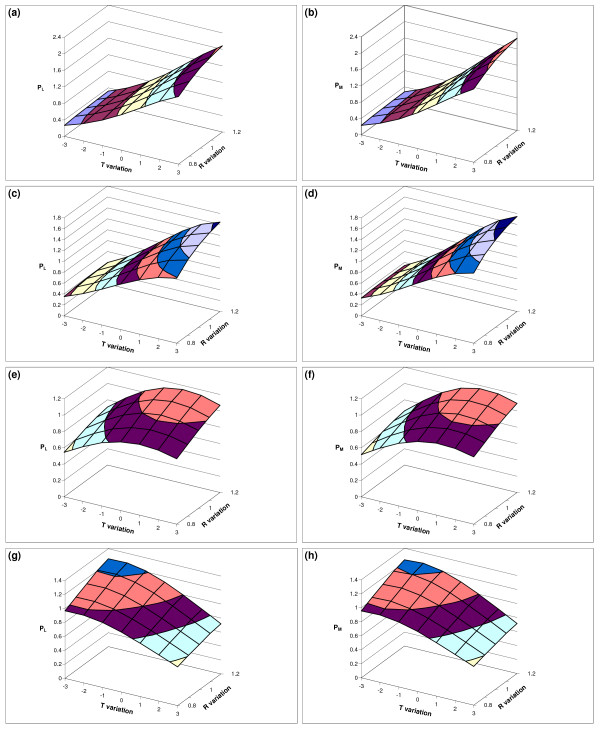
**Results of the sensitivity analysis**. Results of the sensitivity analysis to temperature and rainfall variation. The indexes of variation of population abundance for both immature and adult mosquito are shown for each modified climatic regime (*T *variation × *R *variation). The selected index represents the abundance variation for immature (left) and adult (right) with respect to the reference standard, the average value calculated for the original time series of meteorological data. Pictures (a),(b) refer to the Nairobi site, (c),(d) to Nyabondo, (e),(f) to Kibwesi, (g),(h) to Malindi.

**Nairobi **(1778 *m *asl, yearly mean temperature = 18.8°C, yearly mean rainfall = 796 *mm*). Low temperatures are the main limiting factor for the vector population in this site. If only the effect of temperature is considered (*R*(1) precipitation scenario), population abundance increases with temperature, and the most effective increase is observed in the scenario *T*(+3) with +94% for the adults and +111% for immature (Figure 5a, b). Temperature decrease reduces the mosquito population abundance and the decline reaches -70% for the immature and -74% for the adults for *T*(-3). On the other hand, if only rainfall variations are considered (*T*(0) temperature scenario), rainfall increase results in a much smaller change in the mosquito population (+6% for both immature and adults in the transition from scenario *R*(1) to the *R*(1.2)), while a rainfall decrease leads to a lowering of -14% for immature and -12% for adults in transition from *R*(1) to *R*(0.8).

The variation of the system response to temperature, obtained averaging the effects of the whole range of rainfall levels, follows an almost linear positive trend for both immature and adults, with an average increase of +27.5% and +31% per°C respectively. A similar trend has been obtained for rainfall, although the slope of the interpolating line is much lower (about +5% for both immature and adults per 10% of rainfall increase).

**Nyabondo **(1,532 *m *asl, yearly mean temperature = 20.7°C, yearly mean rainfall = 1,285 *mm*). Thermal influences on vector population are still important in this site but slightly less evident than in Nairobi (Figure 5c, d). If only the effect of temperature is considered ((*R *(1) precipitation scenario), the transition from *T*(0) to *T*(+3) gives a positive change of +50% for immature and +61% for adult population abundance. Vice-versa the strong decrease in temperature *T*(-3) leads to a decrease in abundance by -61% and -66% for immature and adults respectively. The effect of rainfall variation, setting constant the temperature, is similar to that observed in Nairobi, with an increase up to +6% for the immature and +5% for the adult in the transition from *R*(1) to *R*(1.2) and a decrease of -17% and -15% for the same stages in the transition from a *R*(1) to *R*(0.8). Averaging the effects of the whole range of rainfall levels at each temperature, population abundance variation is almost linear and positive with temperature, with a slope of 19% per°C for the immature and 22% for the adults. Also rainfall modification (transition from *R*(0.8) to *R*(1.2)) at different temperatures shows a linear positive trend, with +5.8% and +5.3% for immature and adults every 10% change in precipitation.

**Kibwezi **(904 *m *asl, yearly mean temperature = 23°C, yearly mean rainfall = 712 *mm*). The pattern of variation for this site is quite peculiar, with a clear non-linear, bell-shaped, response surface. The optimal temperature is quite close to the standard conditions, more precisely in the regime *T*(+1) (Figure 5e, f). Most of the departure from the current climate produces a decrease in vector abundance. In *T*(-3) and *R*(1), the reduction is about -35% and -39% for immature and adults respectively. In *T*(+3) and *R*(1) the decrease is less important, reaching -5% for the immature and -2% for the adults. The simulated response of vector to rainfall variation only is quite linear, with an average increase of about 6% for each 10% of rainfall increase for both stages (adults and immature).

**Malindi **(5 *m *asl, yearly mean temperature = 26.9°C, yearly mean rainfall = 889.3 *mm*). The modelled trend of population variation shows that the site is located in a temperature range significantly sub-optimal due to high temperatures (Figure 5g, h). Only negative changes in temperature result in an increase of population abundance. The transition from *T*(0) to *T*(-3) for *R*(1) determines an increase of +12% in the immature and +11% in the adults while the transition from *T*(0) to *T*(+3) for *R*(1) a decrease of -36% for both the immature and the adults. The response surface is nonlinear, particularly if the reduction in temperature is considered. The increase in rainfall produces an almost linear increase of the vector abundance. The increase of population from *R*(0.8) to *R*(1.2), averaging the simulated values at different temperatures, is +6% every 10% of change in precipitation.

### General pattern

**Figure 6:**
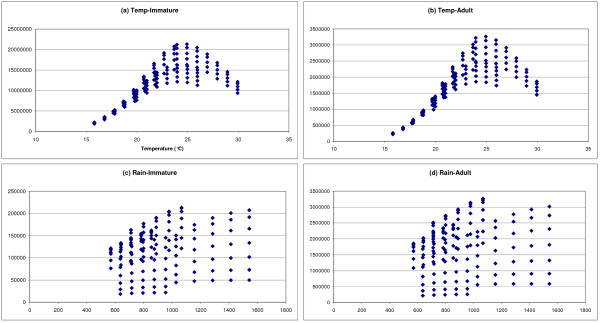
**Simulated average abundance of mosquitoes per cell as function of variation in temperature and rainfall**. Both thermal effects on immature (a) and adult (b) and rainfall effects on immature (c) and adult (d) are shown combining the results obtained for the four selected sites.

The simulated data obtained for different sites and combinations of thermal-pluviometric data have been grouped to evaluate the overall system response to climate variability (Figure 6). Since several sites are taken into account, no specific reference values can be used to normalize the data that are here considered in their absolute values as number of individuals per cell.

The trends of the response variable in Figure 6 show that change in both temperature and rainfall influences the behaviour of the simulated biological system. A clear pattern of variation has been highlighted for the investigated thermal regimes. The mean population abundance follows a bell-shaped distribution with an optimal response for a mean air temperatures of 24-25°C, and a reduction in the abundance of adult and immature population for each departure from this optimal temperature.

The response pattern to rainfall is much less compact, though it is clear that an increase in rainfall always gives an increase (mostly linear) of the vector population abundance. However, the rate of change of the response variable is smaller than the one obtained for air temperature. Therefore, in the range of values analyzed in this paper the system is less sensitive to rainfall variations than to the thermal ones.

## Discussion

Demographic rates of arthropods vectors of human pathogens, as poikilotherm organisms, are sensitive to changes in temperature and, in many cases, also in water availability [39]. The proposed model approaches by a mechanistic point of view the causal chain linking environmental forcing variables to bio-demographic rates and population dynamics of *An. gambiae *s.s. The model allowed to perform a sensitivity analysis on the systematic change in temperature and precipitation at four different Kenyan sites characterized by different landscape, hydrology, climate and epidemiological pattern.

The first investigated site is Nairobi, characterized by a tropical climate modified by highlands (climatic zones 6 in the classification scheme of [52]). Temperatures are relatively low and the precipitation regime is characterized by two maxima in April and November. The site is currently classified as not prone to malaria epidemic, but an increase in temperatures is expected to contribute to make the Nairobi area more suitable to malaria [8]. However, the contribution of rising temperature to vector population abundance appears to be important only for substantial temperature changes. In fact, results of our simulation show how the effect of a temperature increase of 1°C on mosquito development, survival and reproduction produces an adult population variation of +37%, that becomes +77% for +2°C, and +111% for +3°C. The contribution of mosquito abundance change to the local epidemiological pattern is quite difficult to infer. In the Nairobi area, a temperature increase could also give a more rapid sporogonic cycle. Biting rate is also expected to be affected by an increase of mosquito adult population abundance. Only a physiological-based mechanistic approach, considering the interaction among vector, pathogen and host, could highlight how such modification in the vectorial component may contribute to a change in the malaria prevalence.

A decrease in temperature could make the Nairobi area even more protected from malaria risk, as a result of the joint effect of temperature on both the key processes of vector population dynamics and maturation time of the parasite. The effect of negative variations in temperature has proved comparatively more important than the positive one, depleting the adult population of 31%, 56%, and 74% for thermal changes of -1, -2, and -3°C respectively. Furthermore, rainfall changes are expected to be much less important than thermal ones, at least in the tested range -20%/+ 20%. In any case, population dynamics display a positive linear pattern of variation with rainfall increase.

Nyabondo shows an equatorial climate modified by the influence of the lake Victoria (climatic zone 9 of [52]). The main climatic characteristics are high temperatures, high precipitation and absence of dry months [42]. The area is prone to malaria epidemic due to abundant rainfall and temperature relatively close to the thermal optimum for biological performance of mosquito population (Figure 6a, b). Simulated scenarios show a gradual change in adult vector abundance with temperature rise, with an increase of 26%, 47% e 69% for +1, +2 and +3°C respectively. Sensitivity to temperature is less than in Nairobi, because the observed temperatures are closer to the optimum (Figure 6). Temperature decrease produces changes comparable with those obtained for Nairobi; such variation falls in the left trait of the distribution in Figure 6, where the slope of an interpolating curve is expected to be maximum. According to the model simulations, rainfall would not be a limiting factor, in agreement with values that are already high (1,285 *mm*) and well distributed along the year. In any case also for Nyabondo the abundance shows a direct linear positive correlation with the rainfall.

Kibwesi is prone to malaria epidemic and shows a tropical continental/semi-desert climate (zone 5 of [52]) with yearly precipitation below 500 *mm*. The pattern of variation of population dynamics is closely related to mean value of temperatures at this site that is very close to the optimum identified in Figure 6a, b. As a consequence, negative changes in temperature and most of the positive ones negatively effect population abundance. The system is sensitive to changes in rainfall, and the magnitude of variation is comparable with the values simulated for other sites, even if rainfall in this site is lower.

Malindi is characterized by the influence of the Indian Ocean that gives rise to a modified equatorial climate (climatic zones 1 of [52]) with high temperatures moderated by land and sea breeze (oceanic effect), very short or no dry season, two main precipitation maxima (May and November) and high humidity throughout the year. The pattern of response is highly dependent on the position of the average temperature with respect to distribution in Figure 6. Mosquito populations in Malindi are negatively affected by the super-optimal thermal conditions and population abundance may suffer of a further increase in temperature, while it may benefit from a temperature decrease. The gain in population abundance is proportional to the decrease of temperature at a rate that gradually decreases approaching optimal temperature.

The analysis here undertaken is site-specific because in this way we were able to feed the model with time series of real meteorological data deterministically perturbed. However, the pattern emerging from our simulations is of general validity (Figure 6a, b), and significantly follows the typical pattern of many biological response functions to temperature [29]. This has two main implications. First, the three main bio-demographic rate functions (development, mortality and fecundity) are shaped in a way that population performance is optimized at a specific temperature and decreases departing from this thermal optimum. This is important for adaptation and influences habitat selection and species distribution. Second, the distribution in Figure 6a, b could be adopted as an index summarizing the integrated effects of temperatures on development, mortality and reproduction. This index expresses the *An gambiae *s.s. population potential productivity as function of average temperature conditions at local level. The index has a maximum at 25°C and non-linearly decreases toward zero approximately at 14-15°C, with a 10% mean rate of variation for each°C of temperature change. A decrease was also highlighted beyond the thermal optimum with a comparable but negative slope (-10%).

Population abundance typically increases linearly with rainfall variation (Figure 6c, d) throughout the whole precipitation range investigated and independently from the selected site. Symptoms of non-linearity, however, appear at the extremes of the studied range, suggesting that non-linear responses could take place outside the tested interval. The relatively uniform response of the system may be explained by the model parameterization. In fact, in all the sites the landscape is characterized by the same larval habitats features. This oversimplification is undoubtedly a limitation to achieve general conclusions, however it supports the idea that rainfall is a limiting factor and that, to some extent, the increase in water availability, in terms of surface for egg laying, promotes positive linear response in population productivity. In most of the cases such a change in population productivity resulted of 5-6% every 10% of precipitation increase.

The outcome of our analysis warns against any simplistic interpretation of the possible role of climatic variability on the malaria eco-epidemiology. In detail, the issue on climate change influences on vector population dynamics raised in our work leads to the arguments hereafter listed and briefly discussed.

*a*) Climate change analysis cannot be limited to the study of the temperature change effects. For many vector-borne diseases an increasing set of evidences show that other weather components, mainly precipitation and other hydrological variables, can significantly contribute to the system response. Furthermore, as discussed in the methodological section, analysis of climatic scenarios should be carried out taking into account that changes in air temperature and precipitation in tropical climates are correlated. More specifically temperature change results in variation in energy available for convective processes. This translates in changes in thunderstorms activity which in its turn can give rise to relevant feedbacks on surface energy balance and thermal regime [53,54]. Biological response functions may further complicate this picture. As in the cases here analyzed, temperature and rainfall variations does not always drive the change in the system in the same direction, and the interaction between different physical and biological components of the landscape can give rise to complex and nonlinear patterns of change.

*b*) The obtained results provide important insight into the link between temperature change and responses of mosquito population dynamics. The presupposition of a linear response of the vectorial component in the malaria system to temperature changes is excessively simplistic. The reaction of population dynamics to temperature variation is non-linear, as expected considering the well know non-linear response to temperature of the demographic rate functions at the basis of population dynamics [29,55,56]. Such non-linearity also envisages a negative change in the population abundance for temperatures above the optimum temperature. This makes the hypothesized phenomenon of biological amplification of temperature effects [26] valid only for a limited range of temperatures. Moreover, even for climates that are more sensitive to temperature rises, as in the case of Nairobi, it is expected a maximum population abundance variation of 30% for every degree of temperature. This estimate is much smaller (one sixth) than the variation reported, for instance, by [10] which provide, on the basis of correlation analysis, an estimated increase of 100% every + 0.5°C.

*c*) The non-linearity in the temperature-dependent response of population dynamics and the correlation between air temperature and precipitation in tropical climates mean that no simple extrapolations can be done linking temperature raise and increase in distribution and abundance of *An. gambiae *s.s. populations. Therefore, projections on population distribution and productivity should be produced only in the light of the local climate as well as the physical and biological characteristics of the landscape involved in the maintenance of suitable habitats for mosquito. Referring to eco-epidemiological approach we also claim that population projections should take a great advantage from the contribution of process-based model simulation instead of relying on simple indexes and correlation analysis. But ultimately the response pattern of the malaria system can not be interpreted only in the light of the physical and biological factors because behavioural, socio-economic, control operation and other public health measures highly influence the spatial and temporal occurrence of the disease.

*d*) From the model simulations we derived a general pattern of temperature- and rainfall-dependent performance of *An. gambiae *s.s. populations productivity. This should help in defining the expected outcomes of climate variation at fine spatial scales, as well as the interpretation of heterogeneous distribution of mosquito and malaria prevalence in many eco-epidemiological contexts [57-59].

Despite the fact that the analysis is performed on a limited time period and for four sites only, nevertheless the proposed scenarios can be considered realistic and generalizable. From a meteorological point of view, results are supported by the fact that (*i*) the imposed daily air temperature variation is limited to about 2 standard deviations (see table 1) which represent a commonly accepted limit for strong anomalies [60], (*ii*) the reference stations selected represent four different climatic regimes for the Eastern African region, (*iii*) the reference period (27 years) is sufficiently long to capture a great part of the inter-yearly climatic variability that characterize the tropical regimes as a result of geographic, astronomic and circulation factors. Furthermore, the temperature-dependence of the bio-demographic rate functions used in the model, based on a literature review on this issue [30], provides biological foundation to the obtained population dynamics.

As a consequence of the above-mentioned elements the space and time domain of applicability of the results are considered relatively wide [50]. To improve consistency and generality of the analyses performed, the following directions of development are of particular interest:

*a*) Improving model parameterization allowing to tackle morphological, pedological and hydrological characteristics of the landscape. By this point of view, the integration of ground measurements and remote-sensed data of land use, geomorphology and presence/time variability of small water reservoirs could be particularly important;

*b*) Obtaining suitable meteorological and hydrological datasets. The selected datasets are not completely satisfying with reference to average distance among stations and percentage of unavailable data. This highlights a possible problem for model management and show the need of a renewed attention to the quality and representativeness of observational data as crucial elements to express founded judgments on the effect of climate state and variability on tropical diseases;

*c*) Extending the analysis to other temporal and spatial scales. In a temporal perspective it might be interesting to focus on particular periods of the year to evaluate the effects of intra-annual variability of temperature and rainfall. Possible objectives of these studies should be, for example, the evaluation of the influence of specific patterns of rainfall and water resources availability on the rates of survival of mosquitoes during dry periods and the rates of re-colonization in areas with high seasonal rainfall variability. The model would also allow to assess the role of extreme and rare events (e.g., long periods of drought or heavy rainfall) or periodic events (e.g., El Niño-La Niña, the monsoon and their interactions) in conditioning mosquito population dynamics. In a spatial perspective it might be interesting to focus on mesoscale and macro-scale patterns.

*d*) Including in the model other malaria system components. The modular organization of the adopted modelling framework allows to gradually expand the model, integrating the modules for pathogen and human host and test their behaviour as well as the whole system responses with respect to climate variability.

## Conclusions

The sensitivity analysis of *An. gambiae *s.s. population dynamics to climate variability shows a clear non-linear temperature-dependent response, in agreement with the non-linear patterns of temperature-response of the basic bio-demographic processes. The dependence to rainfall is positive and linear for the tested range of variation (± 20%), but non-linearity may appear for higher perturbation. Non-linearity in temperature response of mosquito population dynamics highlighted by our model makes the biological amplification of temperature effects valid only for a limited range of temperatures. As a consequence, no simple extrapolations can be done linking temperature rise with increase in mosquito distribution and abundance. Furthermore, population projections based on hypothesized climatic scenarios should be produced only taking into account the physical and biological characteristics of the landscape.

Regarding the possible relevance of our results for the debate on climate change and malaria it is useful to consider that climate change in the inter-tropical area is a complex phenomenon that involves macroscale circulation patterns (e.g. monsoons, Hadley cell, ITCZ, QBO, ENSO) and their relations with meso and microscale phenomena. Whether, for example, there is a general agreement on the fact that the strengthening of the Hadley circulation would significantly increase the mean precipitation on tropical areas whilst the weakening of tropical monsoons would have the opposite effect, much more difficult is to establish the meso and microscale effects of such hypothesized macroscale changes. The consequence is that the right evaluation of the climate change impact on the vector population system in particular, and the malaria system in general, needs a detailed work on climatic scenarios properly validated and evaluated in the light of time series of circulation patterns and meteo-hydrological variables that drive the malaria system. This is clearly beyond the aim of the sensitivity analysis described in this paper. Nevertheless, we hope that the process-based approach here presented and the proved non linearity linking mosquito population performance to temperature variability may indicate how realistic evaluation of the effects of climate variability on the malaria system has to be founded on right methodological assumptions and procedures in a multidisciplinary perspective. To this purpose, an improvement of the presented model considering the pathogen and the human host compartments and their interaction has been implemented by the authors and some preliminary tests have been carried out. The resulting eco-epidemiological model requires an effective calibration and validation in order to become a valid tool supporting a sensitivity analysis of the malaria system to meteo-hydrological variability.

Finally, the potential use of the proposed modelling framework is not limited to theoretical explorations. As known, a better understanding of the association between malaria and environmental variables has led to increased interest for the development of early warning systems [61]. However the role of the abundance of infected vector is not adequately considered in many tools proposed to support management options evaluation and risk mapping [39]. Forecasting and projection models can be useful to provide predictive capacity to public health professionals, helping the design of epidemic scenarios and the assessment of impact of strategies of control and adaptation [62]. In order to deal with these objectives, the model here proposed could profitably evolve into a powerful and flexible tool for the comparative real-time evaluation of management options for both vector control [63] and environmental management [64], not only for tactical purposes but also at strategic and policy levels.

## Competing interests

The authors declare that they have no competing interests.

## Authors' contributions

GG conceived the design of the analysis, developed and parameterized the epidemiological model and interpreted the biological results. LM developed and parameterized the meteo and the hydrological models, produced the climatic analysis and the scenarios, and performed the simulations. Both the authors contributed to draft the manuscript. All authors read and approved the final manuscript.

## Supplementary Material

Additional file 1 **Kenya climate outlook**. The file provides information about main meteorological features of Kenya territory. Click here for file

Additional file 2 **Physical models of water environment**. After a general description of water bodies relevant for this work, this file provides the description of the hydrological model with calibration and validation activities carried out on it and a list of parameters adopted for the calibrated model. The description of the water temperature model is also provided. Click here for file

Additional file 3 **Scale aspects**. The file provides information about space ant time scales useful for the analysis of the malaria system. Click here for file

Additional file 4 **Reference meteorological stations**. The file contains information about meteorological stations which provided data for this work. Click here for file
