# Supplementary material for: Sensitivity of Anopheles gambiae population dynamics to meteo-hydrological variability: a mechanistic approach
Source: Malar J. 2011 Oct 10;10:294. doi: 10.1186/1475-2875-10-294 (PMC3206495; doi:10.1186/1475-2875-10-294)
Supplement: Additional file 1 — Kenya climate outlook. The file provides information about main meteorological features of Kenya territory. [file 1475-2875-10-294-S1.PDF]

ADDITONAL FILE 1
KENYA CLIMATE OUTLOOK

Tropical climates are under the influence of the Hadley cell, a direct convective cell which extends to the whole troposphere (from the surface to 15-18 km of height) triggering the trade winds at surface. This cell shows an ascending branch (the Intertropical Convergence Zone – ITCZ) at the meteorological equator and a descending one at the tropics. ITCZ presents a seasonal latitudinal oscillation with Northward displacement during the boreal summer.

Hadley cell activity is modulated by some periodic phenomena involving the whole troposphere and more specifically (i) the monsoons, seasonally reversing circulation systems driven mainly by the contrast in the thermal properties of the land and sea surfaces, and two phenomena involving some longitudinal West-East cells along the equator, (ii) the El Nino Southern Oscillation (ENSO), a inter-yearly phenomenon which occurs on timescales of some years and is the result of Walker circulation, and (iii) the Madden-Julian Oscillation (MJO), an intra-seasonal phenomenon which occurs on timescales of 30-60 days [1].

Precipitation regime over Kenya is the effect of three main synoptic patterns: latitudinally migrating ITCZ, monsoonal winds and synoptic scale disturbances. Precipitation areas, produced by the interaction of synoptic patterns with mesoscale features, are fed by humidity coming from Indian Ocean, inland areas with active transpiring vegetation and inland surface waters (mainly Victoria and Turkana lakes).

The two monsoonal wind currents that affect Kenya are the warm and dry NorthEast monsoon airstream that occurs during the Northern Hemisphere winter (December-January-February), and the cool and moist SouthEast monsoonal current that takes place in June-July-August. However the SouthEast monsoon does not deposit any widespread rainfall over Kenya since it is subsiding and diffluent over much of the country [2].

The equinoctial phenomena from March to May (the so called “long rains”) and from October to early December (the so called “short rains”) are the result of the action of ITCZ since the NE and

the SE currents converge over Kenya giving rainfall widespread over the country. Synoptic scale frontal disturbances are superimposed to this primary weather pattern and activate convection in presence of adequate moisture supply.

Table 1.1 summarized some basic scale aspects relevant for this work and the block diagram in figure 1.1 illustrates the causal chain that relates meso to microscale meteorological phenomena to macroscale ones and vice-versa.

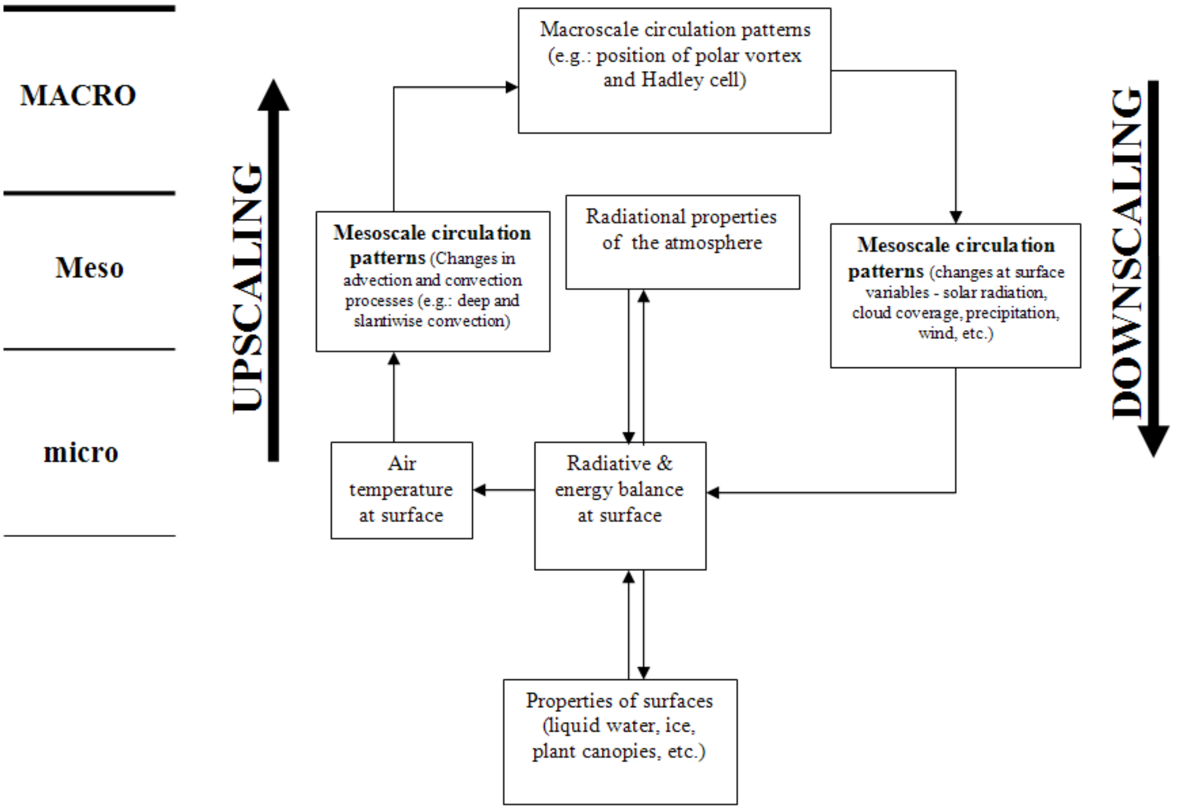

Figure 1.1. *Block diagram illustrating scale relations of the meteorological system.*

Table 1.1. *Space and time scales proposed for the analysis of the malaria system. For each level the spatial and temporal resolution, the type of management, and the main determinants and their characteristic patters are given.*

Reference Level	Spatial resolution	Temporal resolution	Management level	Determinants and characteristic patterns
Macro	100 km	Ten-days	Policy/strategy	Relief (primary mountain ranges)<br>Macroscale circulation patterns like monsoonal winds and latitudinally migrating ITCZ [2].<br>Hydrology of large rivers and lakes [3].<br>Macroscale structure and dynamics of landscape including macro-habitat and human modified ecosystems.<br>Macroscale structure and dynamics of mosquitoes populations in relation to macro-habitat characteristics.<br>Macroscale structure and dynamics of human population.<br>Macroscale temporal and spatial pattern of *Plasmodium* infection and disease epidemiology.
Meso	10 km	Daily	Strategy / tactics	Relief (secondary ranges)<br>Mesoscale circulation patterns like meso-scale systems associated with tropical rainfall patterns or hydrology of little rivers and lakes [3].<br>Mesoscale structure and dynamics of land use (including human settlements).<br>Mesoscale structure and dynamics of mosquitoes populations in relation to land use characteristics.<br>Mesoscale structure and dynamics of human population.<br>Mesoscale dynamics of *Plasmodium* infection and disease epidemiology.
Micro	< 1 km	Hourly	Tactics	Relief (micro-relief)<br>Microscale circulation patterns (e.g.: sea, lake and valley breezes)<br>Microscale patterns of surface meteorological variables (e.g.: precipitation, air temperature)<br>Hydrology of small water bodies (e.g.: ponds, puddles)<br>Fine structure of landscape component at microscale<br>Microscale structure and dynamics of mosquitoes populations in relation to fine land use characteristics<br>Microscale structure and dynamics of human population<br>Microscale dynamics of *Plasmodium* infection and disease epidemiology

References

1. Holton JR: *An introduction to dynamic meteorology*, 4th edition. San Diego: Elsevier

   Academic Press; 2004.

2. Mukabana JR, Pielke RA: **Investigating the influence of synoptic-scale monsoonal winds and mesoscale circulations on diurnal weather patterns over Kenya using a mesoscale circulation model.** *Mon Weather Rev* 1996, **124**:224-243.

3. McKnight TL, Hess D: 2007 *Physical Geography: A Landscape Appreciation. 9th ed.* Upper Saddle River, New Jersey: Prentice-Hall; 2008.
