# Supplementary material for: Sensitivity of Anopheles gambiae population dynamics to meteo-hydrological variability: a mechanistic approach
Source: Malar J. 2011 Oct 10;10:294. doi: 10.1186/1475-2875-10-294 (PMC3206495; doi:10.1186/1475-2875-10-294)
Supplement: Additional file 2 — Physical models of water environment. After a general description of water bodies relevant for this work, this file provides the description of the hydrological model with calibration and validation activities carried out on it and a list of parameters adopted for the calibrated model. The description of the water temperature model is also provided. [file 1475-2875-10-294-S2.PDF]

ADDITIONAL FILE 2
REFERENCE STATIONS AND SCALE ASPECTS

Meteorological stations adopted for this work are listed in Table 2.1 and their spatial distribution is shown in figure 2.1.

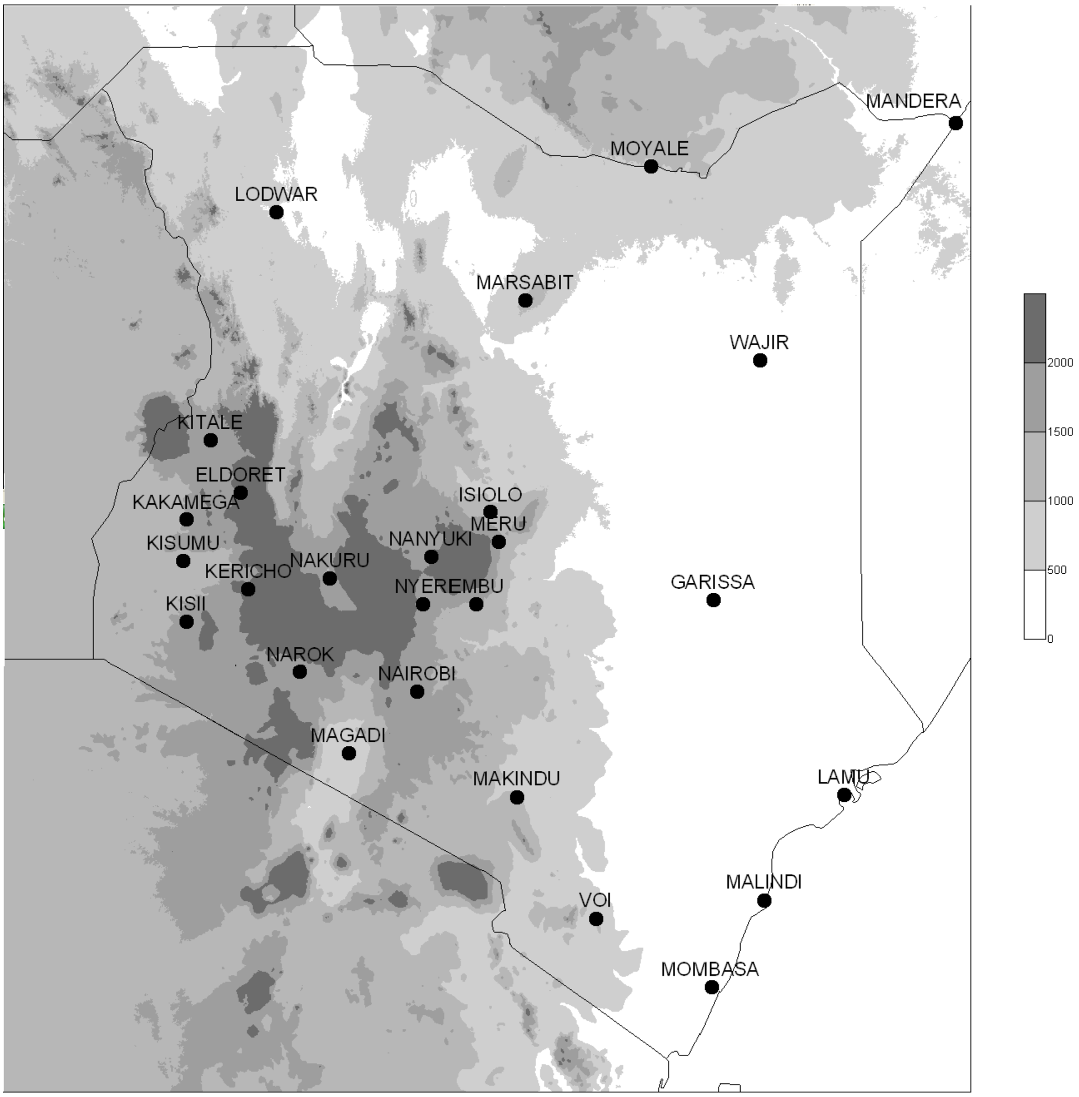

***Figure 2.1** - Spatial distribution of Kenya meteorological stations.*

Table 2.1. *List of the 32 stations used for this work (data from NOAA – Gsod* ftp://ftp.ncdc.noaa.gov/pub/data/gsod/).

N	Code	name	latitude	longitude	height
1	636860	ELDORET	+00.53	+35.28	2133
2	636880	ELDORET/INTERNATION	+00.40	+35.23	2104
3	637200	EMBU	-00.50	+37.45	1493
4	637230	GARISSA	-00.46	+39.63	147
5	637085	ISII	-00.68	+34.70	0
6	636953	ISIOLO	+00.35	+37.58	1067
7	636870	KAKAMEGA	+00.28	+34.78	1530
8	637100	KERICHO	-00.36	+35.35	2184
9	637090	KISII	-00.66	+34.78	1493
10	637080	KISUMU	-00.10	+34.75	1146
11	636610	KITALE	+01.01	+35.00	1875
12	637720	LAMU	-02.26	+40.83	30
13	636120	LODWAR	+03.11	+35.61	515
14	637380	MAGADI	-01.88	+36.28	619
15	637660	MAKINDU	-02.28	+37.83	1000
16	637990	MALINDI	-03.23	+40.10	23
17	636240	MANDERA	+03.93	+41.86	231
18	636410	MARSABIT	+02.30	+37.90	1345
19	636950	MERU	+00.08	+37.65	1554
20	638200	MOMBASA	-04.03	+39.61	55
21	636190	MOYALE	+03.53	+39.05	1097
22	637403	NAIROBI/DAGORETTI	-01.30	+36.75	1798
23	637410	NAIROBI/DAGORETTI	-01.30	+36.75	1798
24	637390	NAIROBI/EASTLEIGH	-01.26	+36.86	1640
25	637400	NAIROBI/KENYATTA	-01.31	+36.91	1624
26	637420	NAIROBI/WILSON	-01.31	+36.81	1679
27	637140	NAKURU	-00.26	+36.10	1901
28	636943	NANYUKI	-00.06	+37.03	1905
29	637370	NAROK	-01.13	+35.83	1890
30	637170	NYERI	-00.50	+36.96	1759
31	637930	VOI	-03.40	+38.56	579
32	636710	WAJIR	+01.75	+40.06	244
