# Supplementary material for: Sensitivity of Anopheles gambiae population dynamics to meteo-hydrological variability: a mechanistic approach
Source: Malar J. 2011 Oct 10;10:294. doi: 10.1186/1475-2875-10-294 (PMC3206495; doi:10.1186/1475-2875-10-294)
Supplement: Additional file 3 — Scale aspects. The file provides information about space ant time scales useful for the analysis of the malaria system. [file 1475-2875-10-294-S3.PDF]

ADDITIONAL FILE 2
EPHEMERAL WATER BODIES OUTLOOK

Ephemeral water bodies commonly occur in depressions without permanent inflow or outflow. Therefore, these pools presents (i) a water balance controlled primarily by direct precipitation, evaporation, and ground-water exchange, though temporary water inflow could be produced by periodic floods, and (ii) an hydroperiod affected by pool morphometry (surface area, volume, depth) and connection to ground water. Hence, the time distribution of rainfall events (amount, intensity, seasonality and frequency/persistence of droughts) is crucial for the persistence of ephemeral water bodies.

Pools supplied only by surface water streams are more ephemeral than those that also receive ground-water inputs. If a pool is not connected to ground water, the rate of net water gain or loss will be equal to the difference between precipitation and evaporation, regardless of pool surface area or basin shape. However, almost all pools have a certain degree of interaction with ground water, which influences the water balance and the hydro-period.

The estimation of water availability in larval habitats is commonly based on the balance between (i) net gains from inflows by direct rainfall and surface water runoff versus (ii) net losses from outflows through soil seepage and percolation [1]. Water balance should take into account morphometric parameters affecting water level and hydro-period, such as depth, area, shape, and volume of pools [2, 3] and hydrologic properties of soils like hydraulic conductivity [4].

The biological characteristics of water bodies are strongly affected by physical driving variables (mainly water temperature and solar radiation) that are the result of energy exchanges with the surrounding (fluxes of sensible and latent heat with atmosphere and ground) driven by the net

radiation fluxes [5]. A dynamic approach to water temperature based on energy balance has been described by [6] and [7].

References

1. Boyd CE, Gross A: **Water use and conservation for inland aquaculture ponds.** *Fisheries Manag Ecol* 2000, **7**:55-63.
2. Brooks RT, Hayashi M: **Depth-area-volume and hydroperiod relationships of ephemeral (vernal) forest pools in Southern New England**. *Wetlands* 2002, **22(2)**:247-255.
3. Porphyre T, Bicout DJ, Sabatier P: **Modelling the abundance of mosquito vectors versus flooding dynamics.** *Ecol Modell* 2005, **183**:173-181.
4. Hill AJ, Neary VS, Morgan KL: **Hydrologic modeling as a development tool for HGM functional assessment models.** *Wetlands* 2006, **26**:161-180.
5. Geiger R: *The climate near the ground*. Cambridge, Mass: Harvard University Press; 1961.
6. van Keulen H, Wolf J (Eds): *Modelling of agricultural production, weather, soils and crops*. Wageningen: Pudoc; 1986.
7. Burba GG, Verma SB, Kim J: **Surface energy fluxes of phragmites australis in a prairie wetland.** *Agr Forest Meteorol* 1999, **94**:31-51.
