# Supplementary material for: Sensitivity of Anopheles gambiae population dynamics to meteo-hydrological variability: a mechanistic approach
Source: Malar J. 2011 Oct 10;10:294. doi: 10.1186/1475-2875-10-294 (PMC3206495; doi:10.1186/1475-2875-10-294)
Supplement: Additional file 4 — Reference meteorological stations. The file contains information about meteorological stations which provided data for this work. [file 1475-2875-10-294-S4.PDF]

ADDITIONAL FILE 4 – THE HYDROLOGICAL MODEL

4.1 ALGORITHM

The hydrological algorithm adopted for this work is hereafter described. The main state variable is the pond water volume while precipitation, runoff, evaporation and seepage are the rate variables. The model algorithm is based on the resolution of the continuity equation applied to water bodies [44]. The following time-discrete equation describes the pond water content *WC*

$$WC(t+1) = WC(t) + Rr + Ru - Ev - Qout - Inf$$

where *Rr* is the daily precipitation, *Ru* is the oncoming stream water from a feeding surface, arbitrarily considered to be 100 times the water body surface, *Qout* is the outgoing stream water and *Inf* is the hourly water infiltration, *Ev* is water surface evaporation, estimated by

$$Ev = C_{Ev} ET0$$

where 1.3 is adopted for the evaporational coefficient C_{Ev} and *ET*0 is the output of Hargreaves and Samani model [62] obtained as

$$ET0 = 0.0023 \sqrt{Tx - Tn} \, (Td + 17.8) \, Ra / 2.45 ,$$

where *Tx*, *Tn*, and *Td* are daily maximum, minimum and mean temperatures, and *Ra* is the extraterrestrial solar radiation [MJ m^{-2} d^{-1}] estimated as a function of latitude by means of standard astronomical equations [62].

Surface runoff *Ru* is estimated using the U.S. Dept. of Agriculture, Soil Conservation Service approach [63]. The estimated runoff *Ru* (mm day^{-1}) is computed using the daily precipitation data measured at the sites

$$Ru = (Rr - 0.2S)^2 / (Rr + 0.8S)$$

where *Rr* is the daily precipitation and *S* is the maximum watershed retention (mm day^{-1}) computed as follows

$$S = (1000 / CN - 10) \, 25.4$$

where *CN* is the runoff curve number obtained from the USSCS table on the base of soil texture, hydrological grouping and land use/vegetation cover [64].

The hourly water infiltration *Inf* is estimated with the Morel-Seytoux equation [65], an approximations of Richards' equation of the water motion in unsaturated soils.

From a geometric point of view, ponds shape is assumed to be a paraboloid of revolution inscribed in a parallelepiped with a squared base (Figure 6). Fulfilled ponds are characterized by a maximum depth $h_{\max}$ and a circular free surface $S_{\max}$ with maximum radius $r_{\max}$. Hence, the total pond volume and surface are is given by

$$V_{\max} = 2\left(h_{\max} r_{\max}^2 \pi\right)/3,$$

$$R_{\max} = r_{\max}^2 \pi \ .$$

The adopted parameterization is characterized by five classes of radius $r_{\max}$ (0.10, 0.25, 0.50, 2 and 5 m). Partially filled ponds show a relation between pond height *h* and radius *r* expressed by the equation $r = h \times shp$, where *shp* is a shape parameter with a constant value of 4.

The pond depth (*h*) variation is the result of the above-mentioned pond water balance. Current values of pond volume (V_{now}) and surface (S_{now}) at each time step are calculated on the base of *h*

$$V_{now} = 2\left(hr^2\pi\right)/3$$

$$S_{now} = r_{\max}^2 \pi$$

hypothesizing the presence of a maximum useful depth for mosquitoes ($h_u = 0.2$ m), for $h \le h_u$ the useful volume V_{util} is equal to V_{now} and useful surface S_{util} is equal to S_{now}; vice versa for $h \ge h_u$

$$r_b = \left(h - h_u\right) shp$$

$V_{\inf} = 2\left(h - h_u\right)/3\, r_b^2 \pi$ (volume of deep water, which is the water at depth greater than maximum useful depth for mosquitoes)

$V_{cyl} = h_u r_b^2 \pi$ (volume of the cylinder that surmounts deep water)

$V_{util} = V_{now} - V_{\inf} - V_{cyl}$

$S_{now} = r^2 \pi$

$S_{\inf} = r_b^2 \pi$

$S_{util} = S_{now} - S_{\inf}$.

4.2 HYDROLOGICAL MODEL PARAMETERIZATION

Table 4.1 shows an output of the pond geometrical model. The general parameterizations adopted for hydrological model are listed in Table 4.2 and specific parameterizations adopted for sensitivity analysis are shown in Table 4.3.

4.3 – CALIBRATION AND VALIDATION

Hydrological model has been calibrated and validated by means of two independent datasets. The first dataset considers data of water level and precipitation reported in [3] and referred to the Tennessee Highland Rim (THR) a depressional wetland located at 86°35'51,99" W and cooy= 35°05’02.36" N, 299 m asl.. Water level data are referred to the period 2003, 8 December – 2004, 8 December and are a continuous series, with the only exception of the period 13 August 14 September with data unavailable. Daily maximum and minimum temperatures are from Nashville station (184 m asl - WMO code 723270). The original depressional wetland (rectangular, about 62x180 m) is represented by an area-equivalent standard pond with radius 60 m and maximum depth of 2.95 m.

The following assumptions has been adopted: (i) inflow of 4 mm/day due to hypodermal runoff assumed for the humid period months (October - December and January-May); (ii) runoff inflow in

rainfall days referred to a surface width 5 times the area of the pond; (iii) saturated hydraulic conductivity of 0.5 mm/h.

The simulated pond water levels follows closely the observed values, as testified by Table 4.4 and Figure 4.1.

For model simulation the following local parameterizations has been adopted: (i) shape of the pond (rectangular, 24 x 8 m) modeled with a standard pond with radius 8 m and maximum depth of 1.01 m, (ii) runoff inflow in rainfall days referred to a surface with the same area of ponds, (iii) Ks of 4 mm/h.

A model verification for equatorial climate has been carried out on data gathered by [4] and referred to an artificial pond established at Kusa (1168 m asl), at the shores of Lake Victoria (Kenya). The run is driven by Tx, Tn, Rr, coming from Kenya GTS stations data interpolated with the IDWM method. The main determinant of Kusa data is the Kisumu station, about 20 km NE of Kusa. Results are presented in Table 4.4 and Figure 4.2. The simulated pond water levels closely correlated with observed values in both cases. Nevertheless two inaccuracies show some peculiar problems of the tropical environment. First, the abrupt increase of water level wrongly simulated on February 9th is the result of a shower of 145.2 mm present in the synthetic time series. This error in precipitation reconstruction is typical of environments with strongly irregular spatial distribution of rainfall. Second, the abrupt increases of pond level on October 27th and December 17th are not simulated because they are the outcomes of floods of the Lake Victoria, a normal phenomenon for the reference location [5].

In conclusion the hydrological model follows pond dynamics with a sufficient realism making possible its use for subsequent sensitivity analysis.

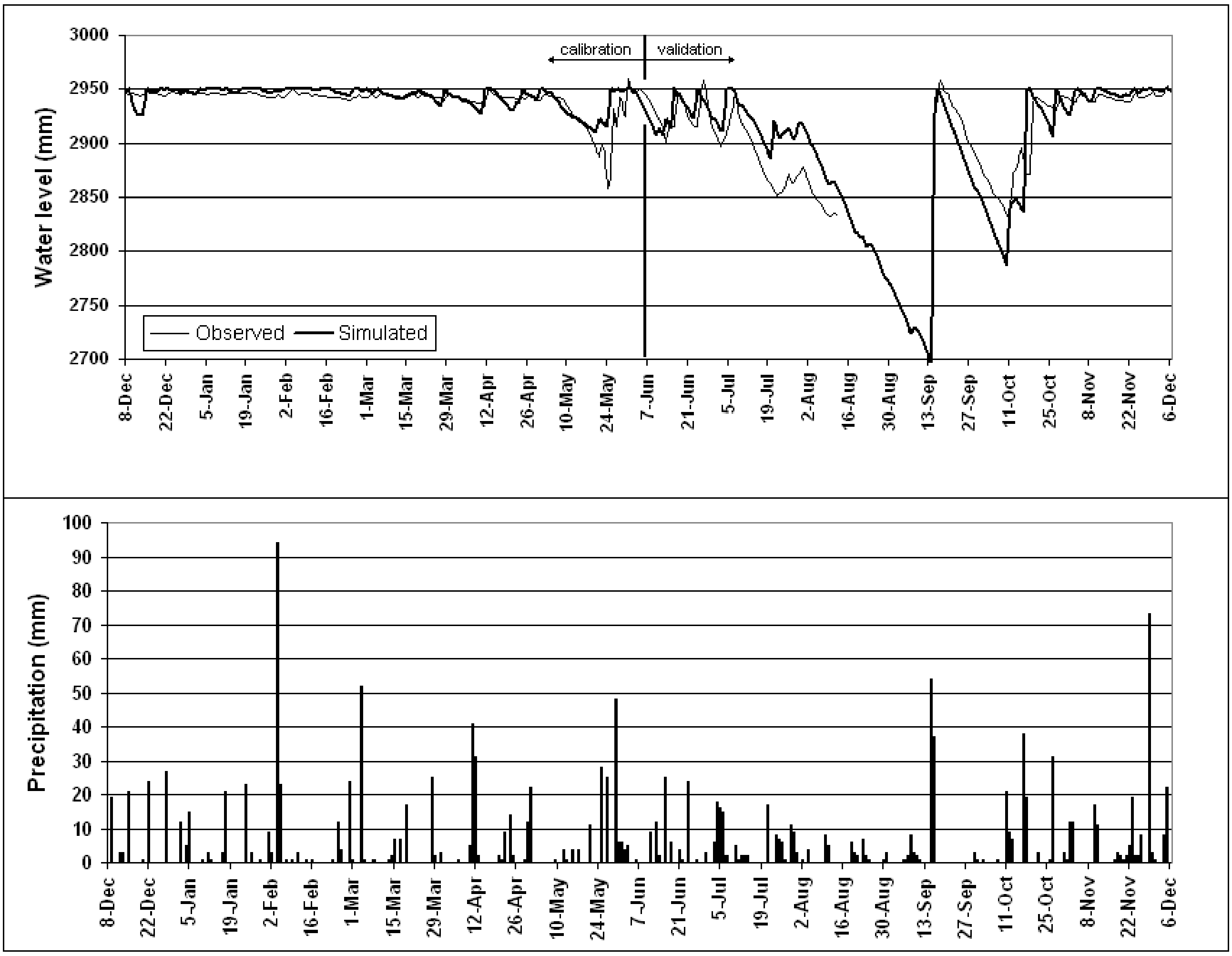

Figure 4.1. *Tennessee Highland Rim (Usa): comparison of simulated and observed water surface elevations from 8/12/2003 to 6/12/2004.*

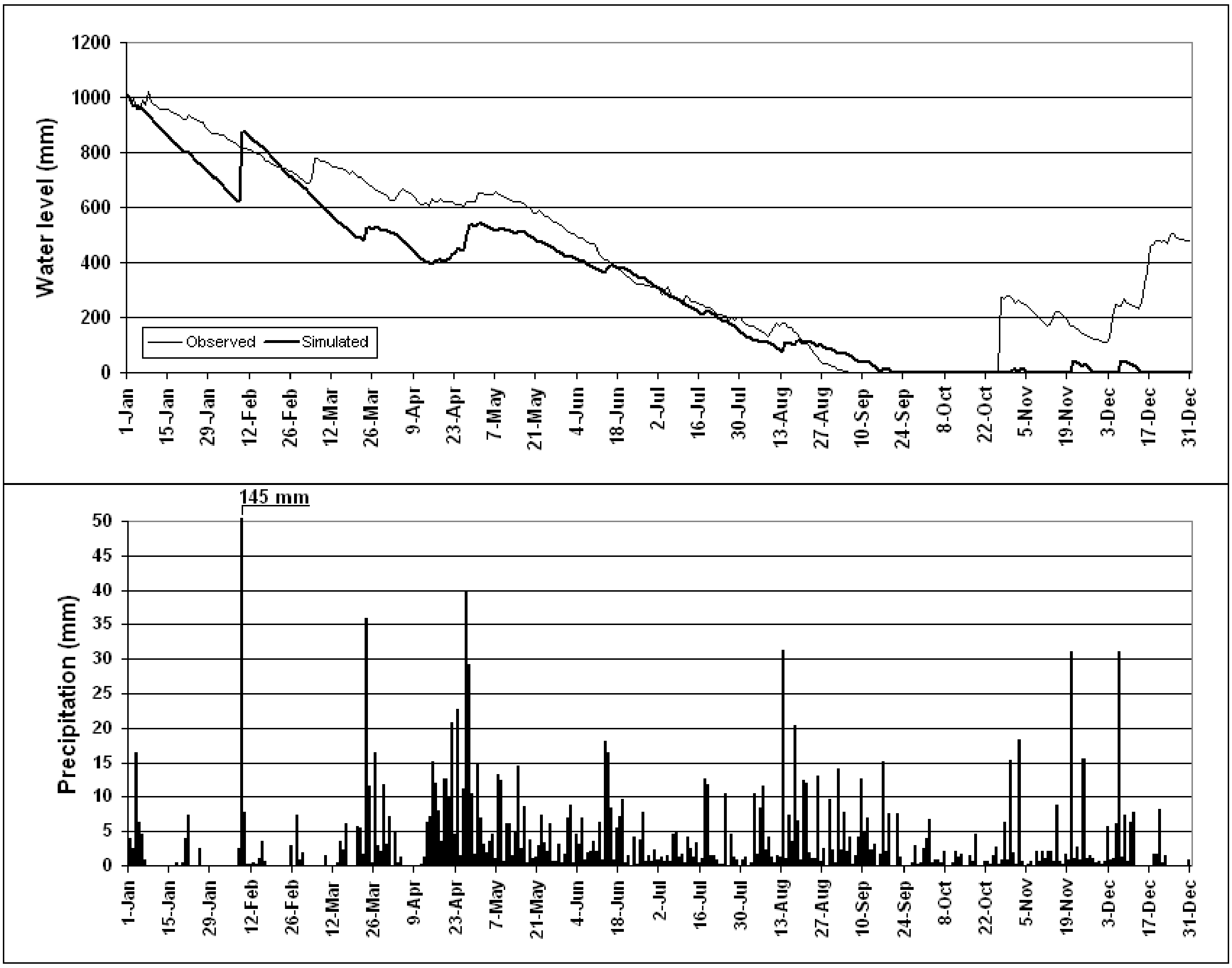

Figure 4.2. *Kusa (Kenya): comparison of simulated and observed water surface elevations from 01/01/2003 to 07/06/2004.*

Table 4.1. *Output of pond geometrical model for ponds with radius of 5 m and with increasing height h.*

H (m)	Vmax (m^3)	Vnow (m^3)	Vutil (m^3)	Vutil/ Vnow (%)	Smax (m^2)	Snow (m^2)	Sutil (m^2)	Sutil/Snow (m^2)
0.1	65.45	0.03	0.03	1.00	78.54	0.50	0.50	1.00
0.2	65.45	0.27	0.27	1.00	78.54	2.01	2.01	1.00
0.3	65.45	0.90	0.77	0.85	78.54	4.52	4.02	0.89
0.4	65.45	2.14	1.47	0.69	78.54	8.04	6.03	0.75
0.5	65.45	4.19	2.38	0.57	78.54	12.57	8.04	0.64
0.6	65.45	7.24	3.49	0.48	78.54	18.10	10.05	0.56
0.7	65.45	11.49	4.79	0.42	78.54	24.63	12.06	0.49
0.8	65.45	17.16	6.30	0.37	78.54	32.17	14.07	0.44
0.9	65.45	24.43	8.01	0.33	78.54	40.72	16.09	0.40
1	65.45	33.51	9.92	0.30	78.54	50.27	18.10	0.36
1.1	65.45	44.60	12.03	0.27	78.54	60.82	20.11	0.33
1.2	65.45	57.91	14.34	0.25	78.54	72.38	22.12	0.31
1.3	65.45	65.45	15.57	0.24	78.54	78.54	23.12	0.29
1.4	65.45	65.45	15.57	0.24	78.54	78.54	23.12	0.29
1.5	65.45	65.45	15.57	0.24	78.54	78.54	23.12	0.29
1.6	65.45	65.45	15.57	0.24	78.54	78.54	23.12	0.29
1.7	65.45	65.45	15.57	0.24	78.54	78.54	23.12	0.29
1.8	65.45	65.45	15.57	0.24	78.54	78.54	23.12	0.29
1.9	65.45	65.45	15.57	0.24	78.54	78.54	23.12	0.29
2	65.45	65.45	15.57	0.24	78.54	78.54	23.12	0.29

Table 4.2. *General parameterizations adopted for the hydrological model. Parameters for the Morel-Seytoux equation have been chosen to obtain a slow and continuous drainage.*

	Value	Units	Reference module	Meaning
β	1.3		Pond water infiltration (Morel-Seytoux model)	Viscous correction parameter introduced by Morel-Seytoux and Khanji [6]. The value of this parameter varies usually between 1 and 1.7 and is generally fixed at 1.3
$\Theta\ s$	0.35	$mm^3\ mm^{-3}$	Pond water infiltration (Morel-Seytoux model)	Volumetric soil water content at saturation (based on field data from [7].
$\Theta\ r$	0.03	$mm^3\ mm^{-3}$	Pond water infiltration (Morel-Seytoux model)	Volumetric residual soil water content (based on field data from [7]) -> In the desaturation zone water drains from the pores and it is displaced by bubbles of air. This stage end at the residual water content $\Theta\ s$ where pore-water becomes occluded (by air) and the permeability is greatly reduced
Hc	100	mm	Pond water infiltration (Morel-Seytoux model)	Capillary height =air entry pressure Hc
$\Theta\ i$	0.10	$mm^3\ mm^{-3}$	Pond water infiltration (Morel-Seytoux model)	Initial moisture conditions AMC at the beginning of the rainfall
Fp	40	mm	Pond water infiltration (Morel-Seytoux model)	Cumulative infiltration before the beginning of ponding.
C_{ev}	1.3	non-dimensional	Pond evaporation coefficient	Multiplicative factor applied to Hargreaves & Samani model [8].
Ks	5.0	mm h-1	Soil saturated hydraulic conductivity	Physical soil property related to infiltration rate [9].
CN	91	non-dimensional	runoff curve number	CN 91 corresponds to the soil group D (clay soils with low infiltration)
Maximum pond depth	2	m		
Runoff_area	15	n times ponds area		
Ev	1	Mm	Water evaporated from surfaces	Water evaporated without reach the soil for precipitation day
AWC	100	Mm	Maximum available water content	Water content between field capacity and wilting point (value for layer explored by roots)

Table 4.3. *Initial conditions and landscape parameterizations adopted for sensitivity analysis.*

model	Parameter	Value	Units
	Size of the cell (spatial unit)	1000000	m^2
**Hydrological**	Classes of ponds	5	number
	Class 1 ponds maximum radius	0.10	*m*
	Class 2 ponds maximum radius	0.25	*m*
	Class 3 ponds maximum radius	0.50	*m*
	Class 4 ponds maximum radius	5.00	*m*
	Class 5 ponds maximum radius	10.00	*m*
	Class 1 ponds number	1200	number per cell
	Class 2 ponds number	100	number per cell
	Class 3 ponds number	50	number per cell
	Class 4 ponds number	2	number per cell
	Class 5 ponds number	1	number per cell
**Vector**	Class 1 ponds – immature individuals	10	individuals per pond
	Class 2 ponds - immature individuals	30	individuals per pond
	Class 3 ponds - immature individuals	40	individuals per pond
	Class 4 ponds - immature individuals	130	individuals per pond
	Class 5 ponds - immature individuals	400	individuals per pond
**Human host**	Total number of human hosts	255	individual per cell

Table 4.4. *Comparison of simulated and observed water surface elevations for Tennessee Highland Rim (USA) and Kusa (Kenya).*

**Test site**	**Variable**	Reference Period	MAE	RMSE	EF	CRM	CD	Slope	Intercept	R2	Significance	Observed Mean	Simulated Mean
Min			0.00	0.00	-inf.	-inf.	0.00	-inf.	-inf.	-inf.			
Max			+inf.	+inf.	1.00	+inf.	+inf.	+inf.	+inf.	+inf.			
Best			0.00	0.00	1.00	0.00	1.00	1.00	0.00	1.00			
Tennessee Highland Rim	Calibration	08-12-2003<br>07-06-2004	5.88	0.35	0.36	0.00	0.56	0.89	321.6	0.41	>99%	2940.4	2943.1
	Validation	08-06-2004<br>06-12-2004	24.52	1.02	0.31	-0.01	0.79	1.30	-894.5	0.71	>99%	2894.0	2915.4
Kusa	Verification	01-01-2003<br>31-12-2003<br>07-06-2004	73.50	28.04	0.89	0.17	0.84	1.08	39.57	0.94	>99%	382.55	317.53
